# Development and Validation of a Predictive Scoring System for Colorectal Cancer Patients With Liver Metastasis: A Population-Based Study

**DOI:** 10.3389/fonc.2021.719638

**Published:** 2021-12-01

**Authors:** Yinghao Cao, Songqing Ke, Shenghe Deng, Lizhao Yan, Junnan Gu, Fuwei Mao, Yifan Xue, Changmin Zheng, Wentai Cai, Hongli Liu, Han Li, Fumei Shang, Zhuolun Sun, Ke Wu, Ning Zhao, Kailin Cai

**Affiliations:** ^1^ Department of Gastrointestinal Surgery, Union Hospital, Tongji Medical College, Huazhong University of Science and Technology, Wuhan, China; ^2^ Wuhan Blood Center, Wuhan, China; ^3^ School of Optical and Electronic Information, Huazhong University of Science and Technology, Wuhan, China; ^4^ College of Life Science and Technology, Huazhong University of Science and Technology, Wuhan, China; ^5^ Cancer Center, Union Hospital, Tongji Medical College, Huazhong University of Science and Technology, Wuhan, China; ^6^ Rizhao City Hospital of Traditional Chinese Medicine (TCM), Rizhao City, China; ^7^ Department of Medical Oncology, Nanyang Central Hospital, Nanyang, China; ^8^ Department of Urology, Third Affiliated Hospital of Sun Yat-sen University, Guangzhou, China

**Keywords:** colorectal cancer, liver metastasis, primary tumpur site, nomogram, overall survival, cancer-specific survival

## Abstract

Liver metastasis in colorectal cancer (CRC) is common and has an unfavorable prognosis. This study aimed to establish a functional nomogram model to predict overall survival (OS) and cancer-specific survival (CSS) in patients with colorectal cancer liver metastasis (CRCLM). A total of 9,736 patients with CRCLM from 2010 to 2016 were randomly assigned to training, internal validation, and external validation cohorts. Univariate and multivariate Cox analyses were performed to identify independent clinicopathologic predictive factors, and a nomogram was constructed to predict CSS and OS. Multivariate analysis demonstrated age, tumor location, differentiation, gender, TNM stage, chemotherapy, number of sampled lymph nodes, number of positive lymph nodes, tumor size, and metastatic surgery as independent predictors for CRCLM. A nomogram incorporating the 10 predictors was constructed. The nomogram showed favorable sensitivity at predicting 1-, 3-, and 5-year OS, with area under the receiver operating characteristic curve (AUROC) values of 0.816, 0.782, and 0.787 in the training cohort; 0.827, 0.769, and 0.774 in the internal validation cohort; and 0.819, 0.745, and 0.767 in the external validation cohort, respectively. For CSS, the values were 0.825, 0.771, and 0.772 in the training cohort; 0.828, 0.753, and 0.758 in the internal validation cohort; and 0.828, 0.737, and 0.772 in the external validation cohort, respectively. Calibration curves and ROC curves revealed that using our models to predict the OS and CSS would add more benefit than other single methods. In summary, the novel nomogram based on significant clinicopathological characteristics can be conveniently used to facilitate the postoperative individualized prediction of OS and CSS in CRCLM patients.

## Introduction

Colorectal cancer (CRC) is the third most common cause of cancer-related death worldwide, with an estimated 145,600 new cases diagnosed in 2019; population-based studies have shown that about 30%–55% of CRC patients develop liver metastasis during their course of the disease (1, 2). In recent years, indications for the treatment of colorectal cancer liver metastases (CRCLM) have expanded, and surgical resection is the only chance of long-term survival. The goal of surgery should be to remove all metastases with negative histological margins while preserving sufficient functional liver parenchyma. Despite advances in oncology and surgery, treatment options vary among individuals, with only about 25% of patients benefiting from them (3, 4).

Predicting long-term survival in patients with CRCLM is a challenge due to genetic, ethnic, dietary, and geographical differences. However, accurate prediction of prognosis is critical for treatment selection and communication between doctors and patients. Previous studies have reported that important predictors of survival in CRCLM patients include multiple liver metastases, positive primary nodules, degree of primary tumor differentiation, extrahepatic spread, tumor size, CEA, positive surgical margin, venous infiltration, and tumor emergence (5, 6). At present, some studies have also found that the survival prognosis of CRCLM patients is closely related to the location of the primary tumor, lymphatic invasion, size and number of hepatic metastases, and the location of the metastatic tumors (7–10). In patients with CRCLM, right lobe metastasis is associated with poorer overall survival (OS) compared with the left lobe; central liver metastasis is a poor prognostic factor after hepatectomy and is associated with early recurrence. Intrahepatic lymphatic invasion, especially combined with vascular invasion, is also an important adverse prognostic factor for OS in patients with single CRCLM after hepatectomy. However, these previous studies did not further evaluate these prognostic risk factors in a comprehensive and systematic manner and study their combinations with the aim of establishing a corresponding prediction model that could accurately predict the prognosis of patients with colorectal cancer.

In our study, information on CRCLM patients was gathered from the SEER database and Wuhan Union Hospital Cancer Centre (WUHCC) cohorts. First, we divided the cases from the SEER database into a training set and an internal validation set. Then, we established an accurate and effective prediction nomogram for OS and cancer-specific survival (CSS) of CRC patients with liver metastasis based on common clinicopathological features and to further develop a histogram to evaluate the predictive value of the prediction nomogram.

## Materials and Methods

### Patients and Study Design

**Figure 1:**
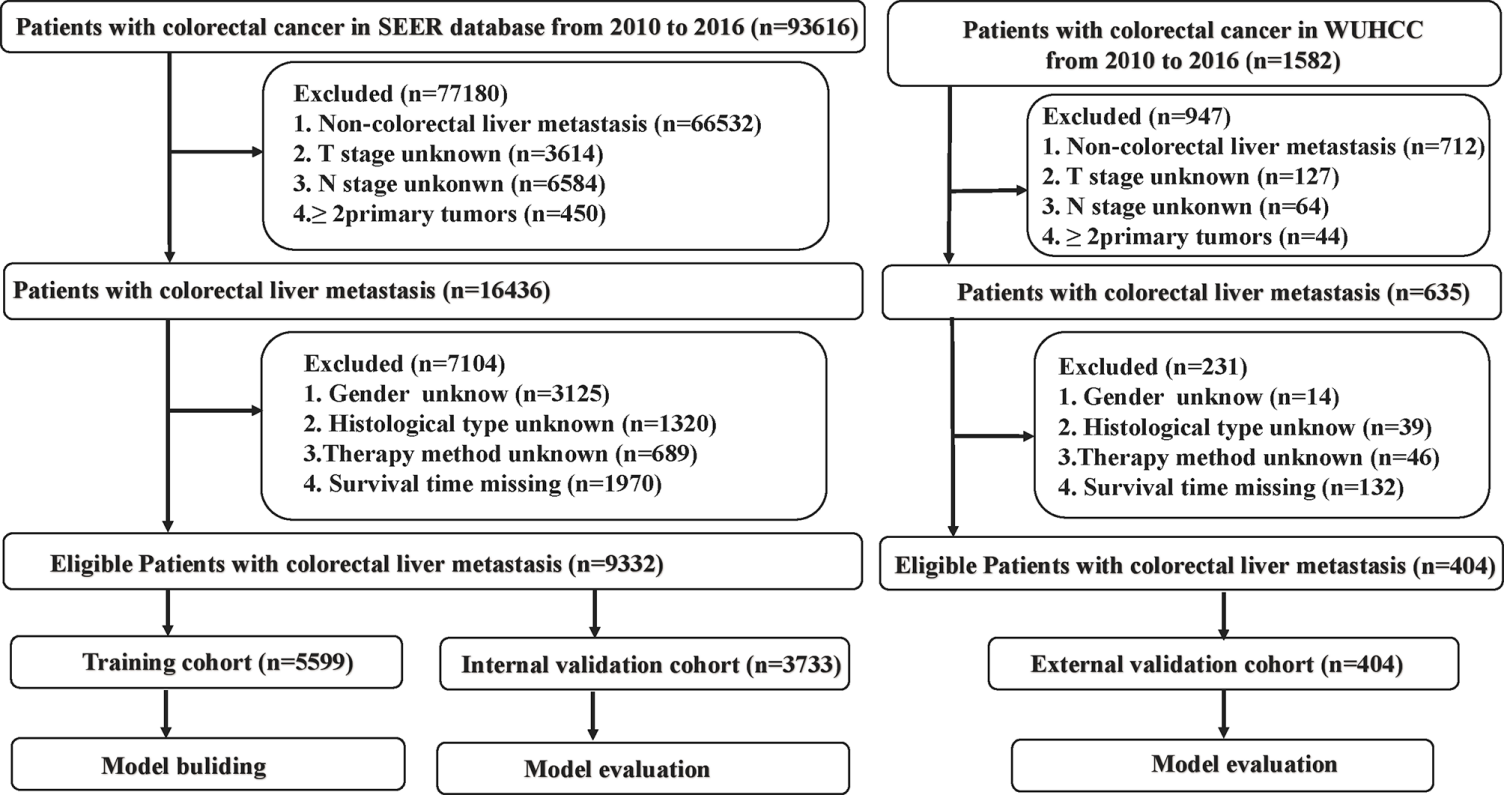
Strategies for selecting patients to be included in the study.

In this study, a total of 9,332 eligible patients from the SEER dataset and 404 patients from the WUHCC cohort with CRCLM were acquired. Selection criteria included the following: CRC patients diagnosed with liver metastasis from 2010 to 2016 of all ages. To identify patients with metastatic CRC cancer to the liver, we selected cases with CRCLM at first diagnosis for further research. Patients who were diagnosed *via* autopsy or death certificate or whose detailed information was unknown or blank were excluded. The detailed flow diagram of the patient selection process is shown in **Figure 1**. Analysis of the data from the SEER program was exempt from medical ethics review, and no informed consent was required. The Ethical Committee and Institutional Review Board of the Wuhan Union Medical College Hospital reviewed and approved this study protocol. All patients provided written informed consent, and all procedures performed in studies involving human participants were in accordance with the Helsinki Declaration.

### Data Collection

Fourteen variables were extracted in this study, including age, gender, tumor location, differentiation, histological type, tumor size, TNM stage, T stage, N stage, M stage, radiotherapy, chemotherapy, number of sample lymph nodes, number of positive lymph nodes, primary surgery, and metastatic surgery. All clinicopathological factors were classified according to the seventh edition of the American Joint Committee on Cancer staging system. The histological type of CRC patients was identified by the International Classification of Diseases for Oncology, 3rd edition (ICD-O-3). According to ICD-O-3 oncology codes, three histological subtypes of CRCs were classified as follows: adenocarcinoma (8010, 8020, 8140–8144, 8210, 8211, 8255, 8260–8263, 8310, 8440, 8460, 8550, 8560), mucinous adenocarcinoma (8470–8472, 8480, 8481), and signet ring cell adenocarcinoma (8490). Patient survival information was measured by CSS and OS. Finally, patients from the SEER database were divided into training and internal validation sets for model building and evaluation. Eligible patients from the WUHCC cohort were used as the external validation cohort for model validation.

### Construction and Validation of the Nomogram

Univariable and multivariable Cox regression analyses were used to calculate the effect of variables on CSS and OS in the training, internal validation, and external validation cohorts. The measure of the effect of each variable on CSS and OS is presented as the hazard ratio (HR) and was used to identify independent risk factors. The variables with *P <*0.05 in the univariate model were used in the multivariate logistic regression analysis. Based on the multivariable Cox regression analysis results, a nomogram integrating clinicopathological parameters was formulated. The overall points for each patient in the training, internal validation, and external validation cohorts were calculated using the established nomogram, after which a Cox regression analysis of the entire cohort was carried out using the overall points as a parameter.

### The Calibration Curve and Area Under the Receiver Operating Characteristic Curve

The calibration of the nomogram was evaluated by the Hosmer–Lemeshow test and displayed in the form of the calibration curve. The accuracy of the predictive ability of the nomogram over time is displayed in the form of 1-, 3-, and 5-year ROC curve, and the discriminative ability of the nomogram to predict OS and CSS in CRCLM is quantitatively expressed by the AUROC.

### The Concordance Index and Decision Curve Analysis

The concordance index (C-index) was defined as the ratio of all patient pairs predicted to be consistent with the results. Decision curve analysis (DCA) was recently proposed as a fresh method to visualize the potential clinical value of a risk prediction model. Thus, the DCA method was carried out to compare the clinical consequences of the predictive nomogram in the current research.

### Statistical Analysis

The categorical variables were expressed as number and percentage, and the chi-square test or Fisher’s exact test was used for comparison where appropriate. Univariable and multivariable Cox regression analyses were used to calculate the effect of variables on CSS and OS. The effect of each variable was evaluated by HR. Stepwise selection was applied to filter variables in the multivariable Cox regression model. The selection criteria were as follows: significant level for entry is 0.05 and the significant level for stay is 0.05. Statistical analysis was conducted with SAS 9.4 (SAS Institute Inc., Cary, NC, USA) and R3.6.2 (R Foundation for Statistical Computing, Vienna, Austria). The R statistical packages “rms”, “survival”, “Hmisc”, “MASS”, and “timeROC” were used to build a nomogram, plot calibration, and time-dependent ROC curves, while “rmda” was used to draw the DCA curves. All statistical tests were two-sided, with statistical significance set at 0.05.

## Results

### Clinicopathological Characteristics and Prognosis of Patients

A total of 9,332 patients from the SEER dataset and 404 patients from the WUHCC cohort with CRCLM between 2010 and 2016 were included in our study. In the SEER database, 60% (*n* = 5,597) of the patients were randomly assigned to the training cohorts, while the remaining patients (*n* = 3,735) were included in the internal validation cohorts. Patients (*n* = 404) from the WUHCC cohort were included in the external validation cohort, and the detailed flow diagram of the patient inclusion and exclusion criteria process is shown in **Figure 1**.

The mortality was 69.9% (41.8% for TVA stage and 26.3% for TVB stage) and 61.6% (38.4% for TVA stage and 21.0% for TVB stage) for all CRCLM patients in the SEER and WUHCC cohorts, respectively. Most patients were older than 60 years (5,313; 56.9%) and male (5,123; 54.9%) in the SEER cohorts and (222; 54.9%; 219; 54.2%) in the WUHCC cohorts, respectively. In both the SEER and WUHCC cohorts, compared with alive patients, dead patients were more likely to have poor tumor differentiation, larger tumor diameter, poor tumor stage, higher rates of lymph node metastasis, tumors localized more commonly in the colon, and metastatic surgery. Moreover, a total of 8.5% and 11.6% of patients underwent adjuvant radiotherapy in the SEER and WUHCC cohorts, respectively. A total of 72.2% and 74.25% of patients underwent adjuvant chemotherapy in the SEER and WUHCC cohorts, respectively (**Table 1**).

**Table 1:** Clinicopathological characteristics of patients in the training, internal validation, and external validation cohorts [*N* (%)].

Characteristics			SEER			SEER			WUHCC		
			Training cohort		*P*	Internal validation cohort		*P*	External validation cohort		*P*
			Alive	Dead		Alive	Dead		Alive	Dead	
			*N* = 1,689	*N* = 3,910		*N* = 1,117	*N* = 2,616		*N* = 155	*N* = 249	
Age	<60		911 (53.94)	1,491 (38.13)	<0.0001	599 (53.63)	1,020 (38.99)	<0.0001	89 (57.42)	93 (37.35)	<0.0001
	≥60		778 (46.06)	2,419 (61.87)		518 (46.37)	1,596 (61.01)		66 (42.58)	156 (62.65)	
Sex	Male		927 (54.88)	2,151 (55.01)	0.930	643 (57.56)	1,402 (53.59)	0.026	94 (60.65)	125 (50.20)	0.0405
	Female		762 (45.12)	1,759 (44.09)		474 (42.44)	1,214 (46.41)		61 (39.35)	124 (49.80)	
Tumor location	Colon		1,286 (76.14)	3,298 (84.35)	<0.0001	828 (74.13)	2,228 (85.17)	<0.0001	96 (61.94)	214 (85.94)	<0.0001
	Rectum		403 (23.86)	612 (15.65)		289 (25.87)	388 (14.83)		59 (38.06)	35 (14.06)	
Differentiation	Well		72 (4.26)	156 (3.99)	<0.0001	38 (3.40)	69 (2.64)	<0.0001	15 (9.68)	7 (2.81)	0.001
	Moderately		1,273 (75.37)	2,451 (62.59)		835 (74.75)	1,656 (63.30)		110 (70.97)	168 (67.47)	
	Poorly		226 (13.38)	980 (25.06)		172 (15.40)	667 (25.50)		18 (11.61)	59 (23.69)	
	Undifferentiated		44 (2.61)	245 (6.27)		33 (2.95)	162 (6.19)		3 (1.94)	8 (3.21)	
	Unknown		74 (4.38)	78 (1.99)		39 (3.49)	62 (2.37)		9 (5.81)	7 (2.81)	
Histological type	Adenocarcinoma, NOS		1,470 (87.03)	3,300 (84.40)	0.031	998 (89.35)	2,171 (82.99)	<0.0001	139 (89.68)	200 (80.32)	0.0986^a^
	Mucinous adenocarcinoma		98 (5.80)	302 (7.72)		45 (4.03)	223 (8.52)		9 (5.81)	26 (10.44)	
	Signet ring cell carcinoma		6 (0.36)	24 (0.61)		6 (0.54)	17 (0.65)		1 (0.65)	3 (1.20)	
	Other		115 (6.81)	284 (7.26)		68 (6.09)	205 (7.84)		6 (3.87)	20 (8.03)	
Size, mm	<55		1,050 (62.17)	2,042 (52.23)	<0.0001	664 (59.44)	1,373 (52.48)	<0.0001	117 (75.48)	143 (57.43)	0.0002
	≥55		639 (37.83)	1,868 (47.77)		453 (40.56)	1,243 (47.52)		38 (24.52)	106 (42.57)	
TNM stage	IVA		1,293 (76.55)	2,312 (59.13)	<0.0001	857 (76.72)	1,596 (61.01)	<0.0001	120 (77.42)	155 (62.25)	0.005
	IVB		357 (21.14)	1,501 (38.39)		233 (20.86)	956 (36.54)		33 (21.39)	85 (34.14)	
	IVNOS		39 (2.31)	97 (2.48)		27 (2.42)	64 (2.45)		2 (1.29)	9 (3.61)	
T stage	T1		23 (1.36)	17 (0.43)	<0.0001	26 (2.33)	16 (0.61)	<0.0001	4 (2.58)	3 (1.20)	0.0003^a^
	T2		85 (5.03)	85 (2.17)		55 (4.92)	54 (2.06)		8 (5.16)	3 (1.20)	
	T3		1,137 (67.32)	2,173 (55.58)		722 (64.64)	1,415 (54.09)		106 (68.39)	140 (56.22)	
	T4		444 (26.29)	1,635 (41.82)		314 (28.11)	1,131 (43.23)		37 (23.87)	103 (41.37)	
N stage	N0		388 (22.97)	483 (12.35)	<0.0001	271 (24.26)	321 (12.27)	<0.0001	63 (40.65)	50 (20.08)	<0.0001
	N1		723 (42.81)	1,361 (34.81)		494 (44.23)	937 (35.82)		59 (38.06)	80 (32.13)	
	N2		578 (34.22)	2,066 (52.84)		352 (31.51)	1,358 (51.91)		33 (21.29)	119 (47.79)	
M stage	M1a		1,293 (76.55)	2,312 (59.13)	<0.0001	857 (76.72)	1,596 (61.01)	<0.0001	120 (77.42)	155 (62.25)	0.005
	M1b		357 (21.14)	1,501 (38.39)		233 (20.86)	956 (36.54)		33 (21.39)	89 (35.74)	
	M1NOS		39 (2.31)	97 (2.48)		27 (2.42)	64 (2.45)		2 (1.29)	5 (2.01)	
Radiotherapy	Yes		225 (13.32)	244 (6.24)	<0.0001	162 (14.50)	158 (6.04)	<0.0001	27 (17.42)	20 (8.03)	0.004
	None/unknown		1,464 (86.68)	3,666 (93.76)		955 (85.50)	2,458 (93.96)		128 (82.58)	229 (91.97)	
Chemotherapy	Yes		1,506 (89.17)	2,575 (65.86)	<0.0001	993 (88.90)	1,666 (63.69)	<0.0001	132 (85.16)	168 (67.47)	<0.0001
	None/unknown		183 (10.83)	1,335 (34.14)		124 (11.10)	950 (36.31)		23 (14.84)	81 (32.53)	
No. of sampled LNs, median (IR)			17 (11)	16 (10)	<0.0001	18 (10)	16 (10)	<0.0001	17 (9)	16 (9)	<0.0001
No. of positive LNs, median (IR)			2 (5)	4 (7)	<0.0001	2 (5)	4 (7)	<0.0001	2 (5)	4 (7)	
Primary surgery		Yes	1,689 (100)	3,906 (99.90)	0.3226	1,117 (100)	2,612 (99.85)	0.324	154 (99.35)	247 (99.20)	1
		No	0	4 (0.10)		0	4 (0.15)		1 (0.65)	2 (0.80)	
Metastatic surgery		Yes	662 (39.19)	956 (24.45)	<0.0001	477 (42.70)	614 (23.47)	<0.0001	40 (25.81)	36 (14.46)	0.0045
		No	1,027 (60.81)	2,954 (75.55)		640 (57.30)	2,002 (76.53)		115 (74.19)	213 (85.54)	

a Fisher.

### Independent Predictive Features in Patients With Colorectal Liver Metastasis

According to the results based on the univariate Cox regression analysis in the training cohort, 13 factors, namely, age, tumor location, differentiation, histological type, tumor size, TNM stage, T stage, N stage, M stage, radiotherapy, chemotherapy, number of sample lymph nodes, and number of positive lymph nodes, were associated with CSS and OS (**Tables 2**, **3**). In the multivariate Cox regression analysis, 10 parameters, including age, tumor location, differentiation, histological type, tumor size, TNM stage, chemotherapy, number of sample lymph nodes, number of positive lymph nodes, tumor size, and metastatic surgery, were independent predictors predicting the OS and CSS for patients with CRCLM (**Tables 4**, **5**).

**Table 2:** Univariate Cox regression model in the training, internal validation, and external validation cohorts of OS.

Variables		Training cohort			Internal validation cohort			External validation cohort		
		HR	95% CI	*P*	HR	95% CI	*P*	HR	95% CI	*P*
Age	<60	1			1			1		
	≥60	1.633	(1.531, 1.743)	<0.0001	1.525	(1.409, 1.649)	<0.0001	1.733	(1.340, 2.241)	<0.0001
Sex	Male	1			1			1		
	Female	1.013	(0.951, 1.079)	0.689	1.15	(1.065, 1.242)	0.0004	1.15	(0.928, 1.526)	0.1698
Tumor location	Colon	1			1			1		
	Rectum	0.684	(0.628, 0.746)	<0.0001	0.606	(0.544, 0.675)	<0.0001	0.428	(0.299, 0.612)	<0.0001
Differentiation	Well	1			1			1		
	Moderately	0.952	(0.809, 1.119)	0.5477	0.944	(0.742, 1.202)	0.6419	1.644	(0.771, 3.504)	0.1979
	Poorly	1.703	(1.438, 2.017)	<0.0001	1.535	(1.198, 1.967)	0.0007	3.46	(1.579, 7.580)	0.0019
	Undifferentiated; anaplastic	2.078	(1.700, 2.541)	<0.0001	1.925	(1.452, 2.551)	<0.0001	9.651	(3.437, 27.098)	<0.0001
	Unknown	0.676	(0.515, 0.887)	0.0048	0.854	(0.606, 1.204)	0.3685	1.897	(0.664, 5.418)	0.2316
Histological type	Adenocarcinoma, NOS	1			1			1		
	Mucinous adenocarcinoma	1.277	(1.135, 1.437)	<0.0001	1.463	(1.275, 1.679)	<0.0001	1,581	(1.049, 2.381)	0.0285
	Signet ring cell carcinoma	2.913	(1.948, 4.356)	<0.0001	1.555	(0.965, 2.507)	0.0698	4.666	(1.475, 14.756)	0.0087
	Other	1.177	(1.043, 1.329)	0.0084	1.29	(1.118, 1.489)	0.0005	1.352	(0.853, 2.142)	0.199
Size, mm	<55	1			1			1		
	≥55	1.319	(1.239, 1.405)	<0.0001	1.217	(1.127, 1.315)	<0.0001	1.529	(1.189, 1.966)	0.0009
TNM stage	IVA	1			1			1		
	IVB	1.759	(1.647, 1.878)	<0.0001	1.787	(1.648, 1.938)	<0.0001	1.66	(1.273, 2.164)	0.0002
	IVNOS	1.523	(1.243, 1.866)	<0.0001	1.299	(1.012, 1.668)	0.0401	1.975	(1.006, 3.878)	0.048
T stage	T1	1			1			1		
	T2	1.24	(0.736, 2.087)	0.4187	1.507	(0.863, 2.633)	0.1493	0.804	(0.162, 3.982)	0.7889
	T3	1.906	(1.182, 3.071)	0.0081	2.208	(1.349, 3.614)	0.0016	1.92	(0.611, 6.031)	0.2639
	T4	3.052	(1.892, 4.923)	<0.0001	3.364	(2.054, 5.510)	<0.0001	3.57	(1.131, 11.272)	0.03
N stage	N0	1			1			1		
	N1	1.339	(1.207, 1.486)	<0.0001	1.447	(1.274, 1.643)	<0.0001	1.187	(0.833, 1.692)	0.3419
	N2	1.987	(1.799, 2.195)	<0.0001	2.051	(1.815, 2.317)	<0.0001	2.1	(1.507, 2.926)	<0.0001
M stage	M1a	1			1			1		
	M1b	1.759	(1.647, 1.878)	<0.0001	1.787	(1.648, 1.938)	<0.0001	1.7	(1.309, 2.208)	<0.0001
	M1NOS	1.523	(1.243, 1.866)	<0.0001	1.299	(1.012, 1.668)	0.0401	1.454	(0.595, 3.551)	0.4112
Radiotherapy	Yes	1			1			1		
	None/unknown	1.756	(1.542, 1.999)	<0.0001	2.072	(1.764, 2.434)	<0.0001	1.846	(1.167, 2.919)	0.0088
Chemotherapy	Yes	1			1			1		
	None/unknown	3.198	(2.990, 3.421)	<0.0001	3.438	(3.169, 3.730)	<0.0001	2.841	(2.169, 3.721)	<0.0001
No. of sampled LNs		0.987	(0.983, 0.990)	<0.0001	0.986	(0.981, 0.990)	<0.0001	0.979	(0.963, 0.997)	0.0188
No. of positive LNs		1.050	(1.045, 1.055)	<0.0001	1.049	(1.042, 1.055)	<0.0001	1.032	(1.012, 1.052)	0.0012
Primary surgery	Yes	1			1			1		
	No	1.672	(0.627, 4.457)	0.3044	1.199	(0.450, 3.198)	0.7166	3.311	(0.819, 13.383)	0.0929
Metastatic surgery	Yes	1			1			1		
	No	1.596	(1.483, 1.717)	<0.0001	1.692	(1.546, 1.853)	<0.0001	1.978	(1.385, 2.825)	0.0002

**Table 3:** Univariate Cox regression model in the training, internal validation, and external validation cohorts of CSS.

Variables		Training cohort			Internal validation cohort			External validation cohort		
		HR	95% CI	*P*	HR	95% CI	*P*	HR	95% CI	*P*
Age	<60	1			1			1		
	≥60	1.595	(1.480, 1.719)	<0.0001	1.539	(1.405, 1.686)	<0.0001	1.844	(1.384, 2.458)	<0.0001
Sex	Male	1			1			1		
	Female	0.991	(0.921, 1.066)	0.8009	1.181	(1.080, 1.291)	0.0003	1.235	(0.938, 1.627)	0.133
Tumor location	Colon	1			1			1		
	Rectum	0.676	(0.611, 0.747)	<0.0001	0.591	(0.521, 0.670)	<0.0001	0.417	(0.279, 0.621)	<0.0001
Differentiation	Well	1			1			1		
	Moderately	1.051	(0.864, 1.278)	0.6212	0.892	(0.679, 1.172)	0.4121	1.893	(0.775, 4.623)	0.1612
	Poorly	1.837	(1.497, 2.253)	<0.0001	1.44	(1.087, 1.908)	0.011	3.789	(1.505, 9.544)	0.0047
	Undifferentiated; anaplastic	2.335	(1.838, 2.967)	<0.0001	1.74	(1.259, 2.406)	0.0008	13.806	(4.421, 43.118)	<0.0001
	Unknown	0.777	(0.567, 1.064)	0.1161	0.808	(0.545, 1.198)	0.2886	1.557	(0.417, 5.809)	0.5099
Histological type	Adenocarcinoma, NOS	1			1			1		
	Mucinous adenocarcinoma	1.289	(1.125, 1.477)	0.0003	1.393	(1.182, 1.641)	<0.0001	1.378	(0.856, 2.217)	0.1871
	Signet ring cell carcinoma	2.496	(1.501, 4.150)	0.0004	1.365	(0.755, 2.470)	0.3065	1.901	(0.264, 13.690)	0.5237
	Other	1.014	(0.872, 1.178)	0.8576	1.301	(1.102, 1.535)	0.0019	1.286	(0.770, 2.149)	0.3366
Size, mm	<55	1			1			1		
	≥55	1.248	(1.160, 1.343)	<0.0001	1.168	(1.068, 1.277)	0.0007	1.544	(1.169, 2.040)	0.0022
TNM stage	IVA	1			1			1		
	IVB	1.799	(1.667, 1.941)	<0.0001	1.749	(1.592, 1.923)	<0.0001	1.856	(1.388, 2.480)	<0.0001
	IVNOS	1.353	(1.052, 1.740)	0.0185	1.199	(0.888, 1.620)	0.2367	2.266	(1.104, 4.649)	0.0257
T stage	T1	1			1			1		
	T2	1.533	(0.811, 2.898)	0.1884	1.229	(0.680, 2.221)	0.494	0.806	(0.163, 3.997)	0.7918
	T3	2.234	(1.235, 4.042)	0.0079	1.833	(1.101, 3.051)	0.0198	1.544	(0.490, 4.865)	0.4586
	T4	3.487	(1.926, 6.314)	<0.0001	2.573	(1.543, 4.289)	0.0003	3.075	(0.970, 9.746)	0.0563
N stage	N0	1			1			1		
	N1	1.412	(1.251, 1.593)	<0.0001	1.474	(1.273, 1.708)	<0.0001	1.268	(0.858, 1.874)	0.2333
	N2	1.994	(1.775, 2.240)	<0.0001	2.056	(1.784, 2.369)	<0.0001	2.049	(1.413, 2.972)	0.0002
M stage	M1a	1			1			1		
	M1b	1.799	(1.667, 1.941)	<0.0001	1.749	(1.592, 1.923)	<0.0001	1.89	(1.419, 2.518)	<0.0001
	M1NOS	1.353	(1.052, 1.740)	0.0185	1.199	(0.888, 1.620)	0.2367	1.858	(0.757, 4.559)	0.176
Radiotherapy	Yes	1			1			1		
	None/unknown	1.843	(1.581, 2.149)	<0.0001	2.008	(1.671, 2.412)	<0.0001	2.046	(1.207, 3.467)	0.0078
Chemotherapy	Yes	1			1			1		
	None/unknown	3.185	(2.945, 3.444)	<0.0001	3.352	(3.047, 3.687)	<0.0001	2.677	(1.977, 3.624)	<0.0001
No. of sampled LNs		0.985	(0.981, 0.989)	<0.0001	0.986	(0.981, 0.991)	<0.0001	0.977	(0.959, 0.996)	0.0196
No. of positive LNs		1.047	(1.041, 1.053)	<0.0001	1.049	(1.042, 1.056)	<0.0001	1.027	(1.004, 1.049)	0.0191
Primary surgery	Yes	1			1			1		
	No	2.305	(0.866, 6134)	0.0945	1.629	(0.611, 4.347)	0.3294	4.236	(1.045, 17.171)	0.0432
Metastatic surgery	Yes	1			1			1		
	No	1.615	(1.483, 1.758)	<0.0001	1.7	(1.530, 1.888)	<0.0001	1.818	(1.242, 2.663)	0.0021

**Table 4:** Multivariable cox regression model in the training, internal validation, and external validation cohorts of OS.

Variables		Training dataset			Internal validation cohort			External validation cohort		
		HR	95% CI	*P*	HR	95% CI	*P*	HR	95% CI	*P*
Age	<60	1			1			1		
	≥60	1.283	(1.200, 1.373)	<0.0001	1.195	(1.102, 1.296)	<0.0001	1.48	(1.131, 1.937)	0.0043
Tumor location	Colon	1.000			1			1		
	Rectum	0.832	(0.762, 0.909)	<0.0001	0.728	(0.652, 0.812)	<0.0001	0.427	(0.294, 0.620)	<0.0001
Differentiation	Well	1.000			1			1		
	Moderately	1.082	(0.920, 1.273)	0.3399	1.043	(0.819, 1.329)	0.7306	1.412	(0.656, 3.042)	0.378
	Poorly	1.564	(1.317, 1.857)	<0.0001	1.489	(1.161, 1.911)	0.0017	2.26	(1.007, 5.073)	0.0481
	Undifferentiated; anaplastic	1.868	(1.523, 2.291)	<0.0001	1.683	(1.267, 2.235)	0.0003	6.371	(2.172, 18.691)	0.0007
	Unknown	0.916	(0.697, 1.204)	0.5292	1.162	(0.823, 1.641)	0.3924	1.036	(0.349, 3.076)	0.9488
Histological type	Adenocarcinoma, NOS	1			1			1		
	Mucinous adenocarcinoma	1.148	(1.019, 1.293)	0.0229	1.132	(0.984, 1.302)	0.0818	1.639	(1.048, 2.564)	0.0305
	Signet ring cell carcinoma	1.589	(1.058, 2.386)	0.0255	1.222	(0.755, 1.980)	0.4141	4.308	(1.312, 14.148)	0.0161
	Other	1.164	(1.030, 1.317)	0.0153	1.064	(0.921, 1.230)	0.3991	1.056	(0.654, 1.705)	0.8224
Size, mm	<55	1			1			1		
	≥55	1.223	(1.147, 1.303)	<0.0001	1.11	(1.027, 1.201)	0.0089	1.272	(0.972, 1.665)	0.0799
TNM	IVA	1			1			1		
	IVB	1.569	(1.467, 1.677)	<0.0001	1.579	(1.453, 1.715)	<0.0001	1.454	(1.101, 1.921)	0.0083
	IVNOS	1.187	(0.966, 1.457)	0.1028	1.188	(0.924, 1.527)	0.1797	2.114	(1.053, 4.245)	0.0352
Chemotherapy	Yes	1.000			1			1		
	None/unknown	2.916	(2.718, 3.129)	<0.0001	3.065	(2.816, 3.336)	<0.0001	2.74	(2.059, 3.647)	<0.0001
No. of sampled LNs		0.977	(0.973, 0.981)	<0.0001	0.979	(0.974, 0.983)	<0.0001	0.961	(0.943, 0.979)	<0.0001
No. of positive LNs		1.053	(1.047, 1.059)	<0.0001	1.049	(1.041, 1.056)	<0.0001	1.026	(1.003, 1.050)	0.025
Metastatic surgery	Yes	1.000			1			1		
	No	1.364	(1.267, 1.469)	<0.0001	1.301	(1.186, 1.428)	<0.0001	1.423	(0.984, 2.058)	0.0612

**Table 5:** Multivariable Cox regression model in the training, internal validation, and external validation cohorts of CSS.

Variables		Training dataset			Internal validation cohort			External validation cohort		
		HR	95% CI	*P*	HR	95% CI	*P*	HR	95% CI	*P*
Age	<60	1			1			1		
	≥60	1.253	(1.160, 1.355)	<0.0001	1.221	(1.111, 1.341)	<0.0001	1.606	(1.194, 2.160)	0.0017
Tumor location	Colon	1			1			1		
	Rectum	0.91	(0.845, 0.980)	0.013	1.102	(1.007, 1.206)	0.0339	1.274	(0.960, 1.693)	0.094
Differentiation	Well	1			1			1		
	Moderately	0.807	(0.728, 0.894)	<0.0001	0.712	(0.626, 0.809)	<0.0001	0.416	(0.275, 0.630)	<0.0001
	Poorly	1			1			1		
	Undifferentiated; anaplastic	1.187	(0.976, 1.445)	0.0867	0.974	(0.741, 1.280)	0.8487	1.549	(0.628, 3.820)	0.3421
	Unknown	1.707	(1.388, 2.099)	<0.0001	1.387	(1.045, 1.840)	0.0235	2.453	(0.955, 6.297)	0.0622
Histological type	Adenocarcinoma, NOS	2.173	(1.706, 2.767)	<0.0001	1.508	(1.088, 2.090)	0.0135	9.22	(2.876, 29.555)	0.0002
	Mucinous adenocarcinoma	1.073	(0.782, 1.473)	0.6605	1.106	(0.745, 1.642)	0.6176	1.006	(0.265, 3.818)	0.9928
	Signet ring cell carcinoma	1			1			1		
	Other	1.167	(1.084, 1.257)	<0.0001	1.08	(0.986, 1.183)	0.0961	1.286	(0.958, 1.727)	0.0941
Size, mm	<55	1			1			1		
	≥55	1.628	(1.506, 1.759)	<0.0001	1.546	(1.404, 1.703)	<0.0001	1.663	(1.226, 2.254)	0.0011
TNM	IVA	1.07	(0.830, 1.379)	0.6022	1.096	(0.810, 1.483)	0.5516	2.356	(1.116, 4.973)	0.0246
	IVB	1			1			1		
	IVNOS	2.953	(2.720, 3.205)	<0.0001	2.99	(2.708, 3.300)	<0.0001	2.573	(1.870, 3.540)	<0.0001
No. of sampled LNs		0.977	(0.972, 0.981)	<0.0001	0.979	(0.973, 0.985)	<0.0001	0.961	(0.941, 0.982)	0.0003
No. of positive LNs		1.051	(1.044, 1.058)	<0.0001	1.049	(1.041, 1.058)	<0.0001	1.02	(0.994, 1.047)	0.1298
Metastatic surgery	Yes	1			1			1		
	No	1.38	(1.266, 1.504)	<0.0001	1.312	(1.179, 1.461)	<0.0001	1.377	(0.930, 2.039)	0.1103

### Construction of the Nomogram

**Figure 2:**
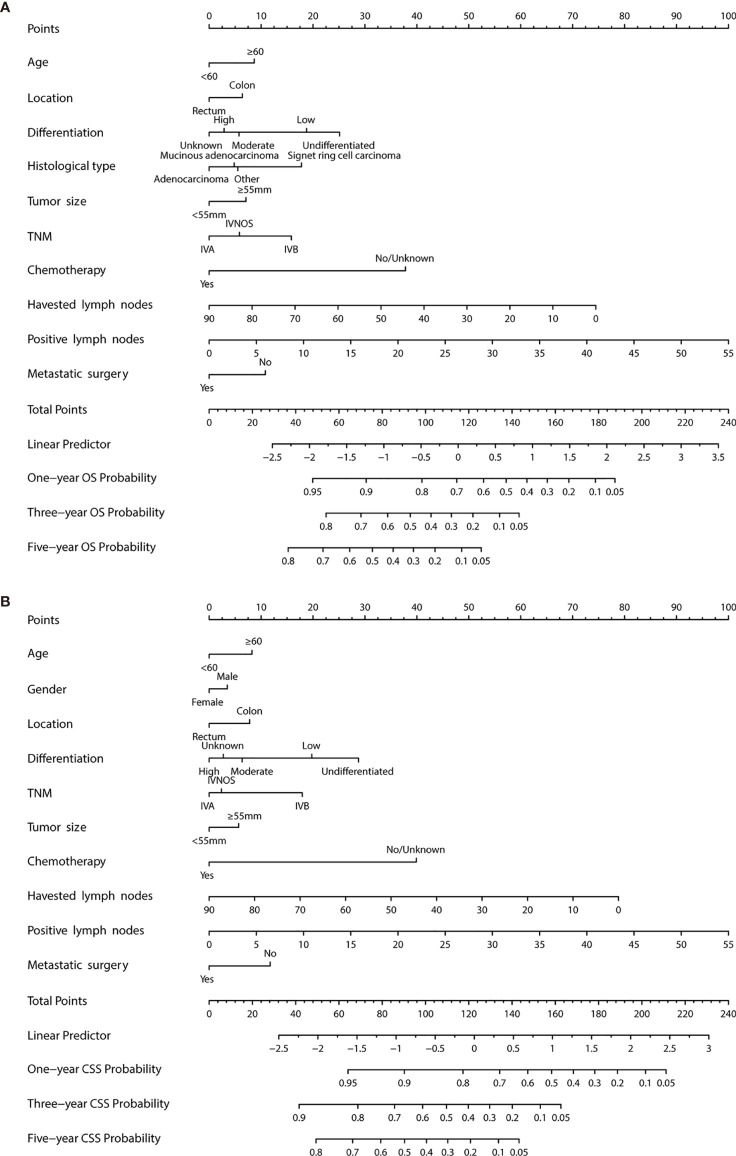
Evaluation of overall survival (OS) and cancer-specific survival (CSS)-associated nomograms for patients with colorectal liver metastasis (CRCLM). **(A)** OS nomogram integrating age, tumor location, differentiation, gender, TNM stage, chemotherapy, no. of sample LNs, no. of positive LNs, tumor size, and metastatic surgery for predicting 1-, 3-, and 5-year OS rates. **(B)** CSS nomogram integrating age, tumor location, differentiation, gender, TNM stage, chemotherapy, no. of sample LNs, no. of positive LNs, tumor size, and metastatic surgery for predicting 1-, 3-, and 5-year CSS rates.

Multivariable Cox regression analysis was used to identify the significant features associated with survival status, based on which the nomogram for OS and CSS was developed. The risk score associated with each variable and projection can be obtained by the top ruler, and the probabilities for 1-, 3-, and 5-year CSS and OS can be estimated by superposing the risk score of each variable to the bottom ruler (**Figures 2A, B**).

### Comparisons of the Nomogram and TNM Stage

**Figure 3:**
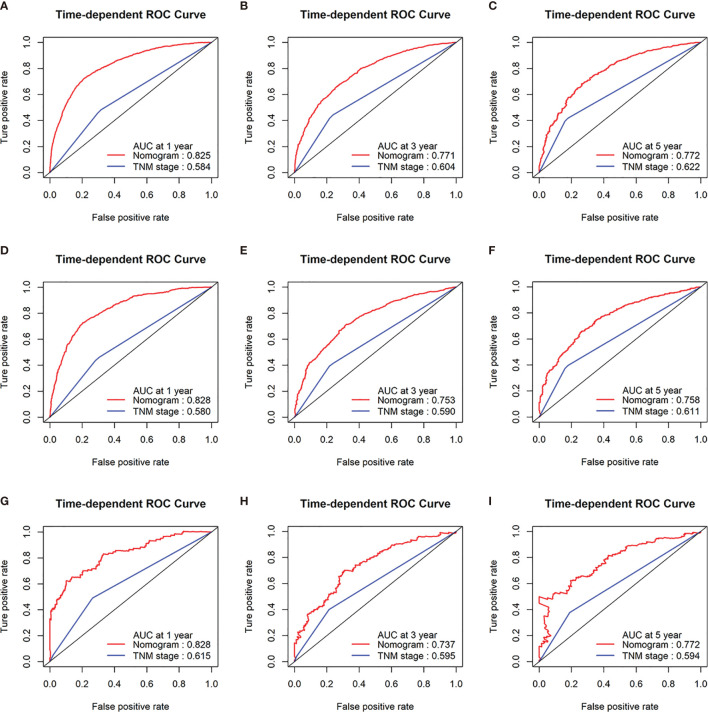
Receiver operating characteristic (ROC) curves of TNM stage and the nomogram of the CSS. AUC values of ROC predicted 1-, 3-, and 5-year CSS rates of the nomogram and TNM stage in the training cohorts **(A–C)**; AUC values of ROC predicted 1-, 3-, and 5-year CSS rates of the nomogram and TNM stage in the internal validation cohorts **(D–F)**; AUC values of ROC predicted 1-, 3-, and 5-year CSS rates of the nomogram and TNM stage in the external validation cohorts **(G–I)**.

To confirm that the nomogram had higher efficacy in predicting the prognosis of patients with CRCLM than TNM stage, time-dependent ROC analyses at 1, 3, and 5 years were conducted. The 1-, 3-, and 5-year AUC values of the nomogram for the prediction of CSS were 0.825, 0.771, and 0.772 in the training cohorts, respectively, compared with 0.584, 0.604, and 0.622, respectively, for the AUC values of TNM stage. The 1-, 3-, and 5-year AUC values of the nomogram for the prediction of CSS were 0.828, 0.753, and 0.758 in the internal validation cohorts, compared with 0.580, 0.590, and 0.611, respectively, for the AUC values of TNM stage. The 1-, 3-, and 5-year AUC values of the nomogram for the prediction of CSS were 0.828, 0.737, and 0.772 in the external validation cohorts, compared with 0.615, 0.595, and 0.594, respectively, for the AUC values of TNM stage (**Figures 3A–I**).

**Figure 4:**
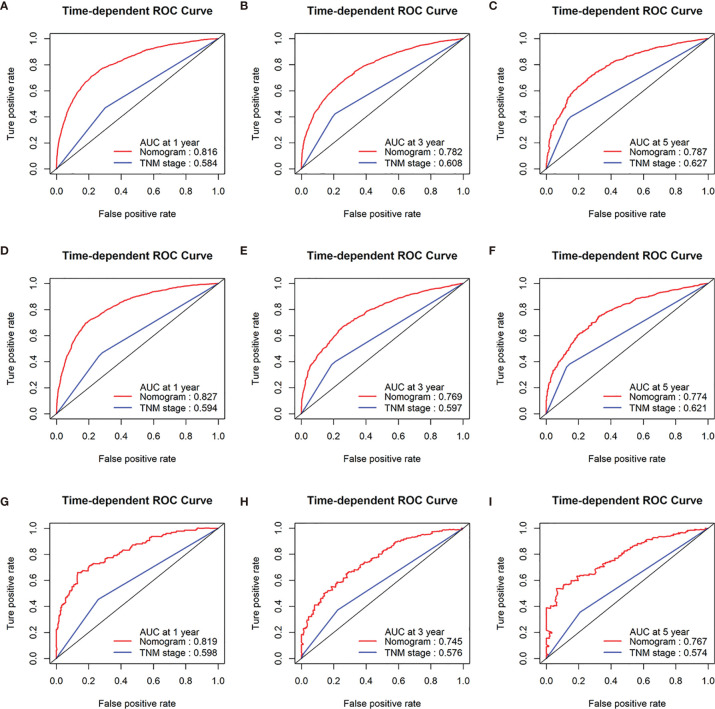
Receiver operating characteristic (ROC) curves of TNM stage and the nomogram of the OS. AUC values of ROC predicted 1-, 3-, and 5-year OS rates of the nomogram and TNM stage in the training cohorts **(A–C)**; AUC values of ROC predicted 1-, 3-, and 5-year OS rates of the nomogram and TNM stage in the internal validation cohorts **(D–F)**; AUC values of ROC predicted 1-, 3-, and 5-year OS rates of the nomogram and TNM stage in the external validation cohorts **(G–I)**.

In addition, the 1-, 3-, and 5-year AUC values of the nomogram for the prediction of OS were 0.816, 0.782, and 0.787 in the training cohorts, respectively, compared with 0.584, 0.627, and 0.608, respectively, for the AUC values of TNM stage. The 1-, 3-, and 5-year AUC values of the nomogram for the prediction of OS were 0.827, 0.769, and 0.774 in the internal validation cohorts, compared with 0.594, 0.597, and 0.621, respectively, for the AUC values of TNM stage. The 1-, 3-, and 5-year AUC values of the nomogram for the prediction of OS were 0.819, 0.745, and 0.767 in the external validation cohorts, compared with 0.598, 0.576, and 0.574, respectively, for the AUC values of TNM stage (**Figures 4A–I**).

### Evaluation and External Validation of the OS and CSS Prediction Nomogram

**Figure 5:**
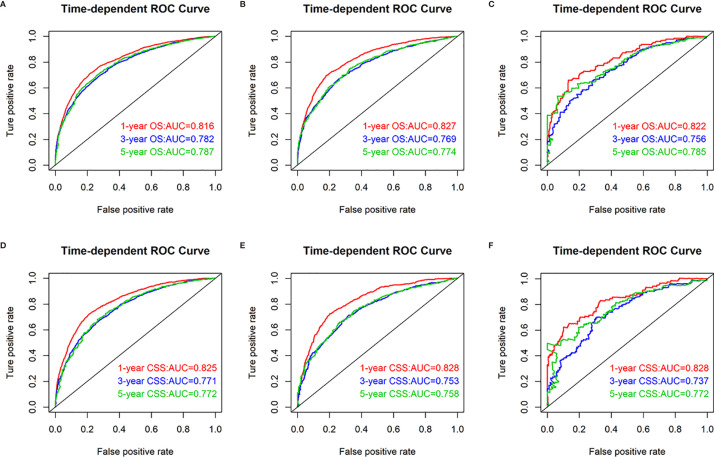
Nomograms of time-dependent receiver operating characteristic (ROC) curves associated with overall survival (OS) and cancer-specific survival (CSS). **(A–C)** Represents AUC values of ROC predicted 1-, 3-, and 5-year OS rates of the nomogram for the training, internal validation, and external validation cohorts; **(D–F)** represents AUC values of ROC predicted 1-, 3-, and 5-year CSS rates of the nomogram for the training, internal validation, and external validation cohorts.

The discrimination ability of the nomogram was represented by the ROC curve. The nomograms showed favorable sensitivity at predicting 1-, 3-, and 5-year OS, with the AUROC values of 0.816, 0.782, and 0.787 in the training cohort, respectively; 0.827, 0.769, and 0.774 in the internal validation cohort, respectively; and 0.819, 0.745, and 0.767 in the external validation cohort, respectively. For CSS, the 1-, 3-, and 5-year predictive power of survival nomograms measured by AUC in the training cohort was 0.825, 0.771, and 0.772, respectively; in the internal validation cohort, it was 0.828, 0.753, and 0.758, respectively; and in the external validation cohort, it was 0.828, 0.737, and 0.772 (**Figure 5**).

C-index values and ROC curves are ordinarily used to evaluate the discriminatory power of a nomogram. The C-indexes for the prediction of CSS were 0.74 (95% CI 0.730, 0.750), 0.746 (95% CI 0.734, 0.758), and 0.755 (95% CI 0.718, 0.792) in the training, internal validation, and external validation cohorts, respectively. In addition, the C-indexes for the prediction of OS were 0.743 (95% CI 0.735, 0.751), 0.748 (95% CI 0.738, 0.758), and 0.756 (95% CI 0.723, 0.789) in the training, internal validation, and external validation cohorts, indicating that the nomogram had favorable discrimination in patients with CRCLM.

**Figure 6:**
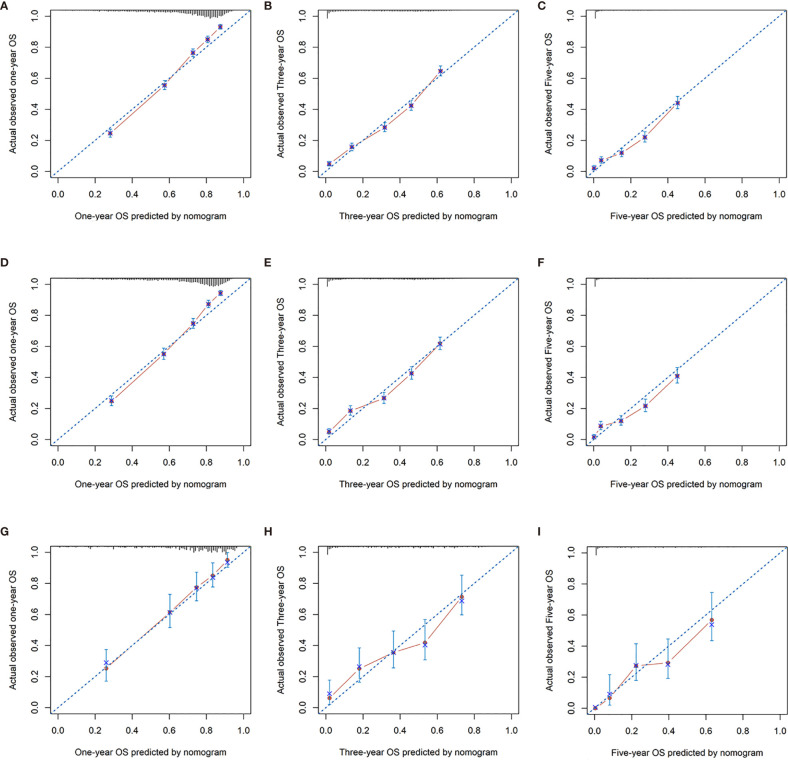
Calibration curves for 1-, 3-, and 5-year OS rate of nomogram predictions. **(A–C)** Represents the calibration curve for predicting the OS of patients at 1, 3, and 5 years in the training cohorts; **(D–F)** represents the calibration curve for predicting the OS of patients at 1, 3, and 5 years in the internal validation cohorts; **(G–I)** represents the calibration curve for predicting the OS of patients at 1, 3, and 5 years in the external validation cohorts.

**Figure 7:**
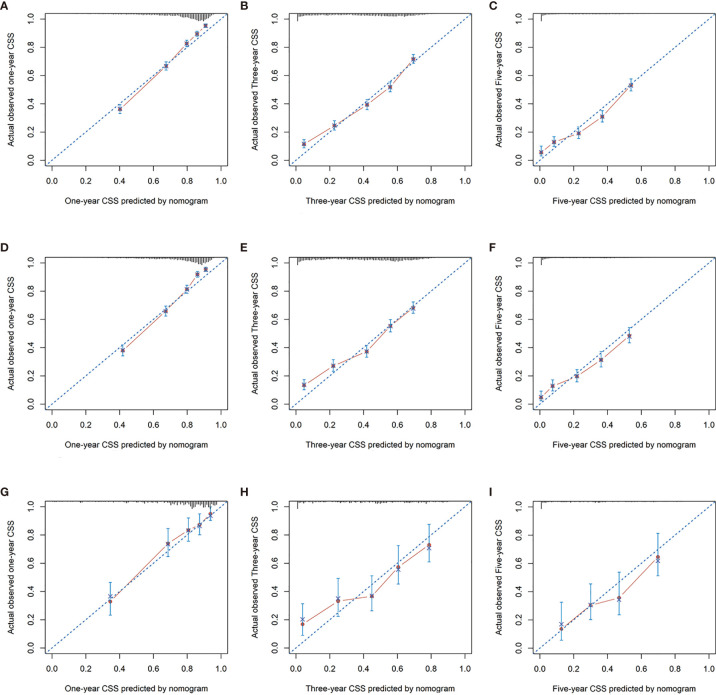
Calibration curves for 1-, 3-, and 5-year CSS rate of nomogram predictions. **(A–C)** Represents the calibration curve for predicting the CSS of patients at 1, 3, and 5 years in the training cohorts; **(D–F)** represents the calibration curve for predicting the CSS of patients at 1, 3, and 5 yeara in the internal validation cohorts; **(G–I)** Represents the calibration curve for predicting the CSS of patients at 1, 3, and 5 years in the external validation cohorts.

In addition, calibration curves for the nomogram showed no deviations from the reference line, indicating a high degree of credibility (**Figures 6**, **7**).

### Clinical Value of the Nomogram

**Figure 8:**
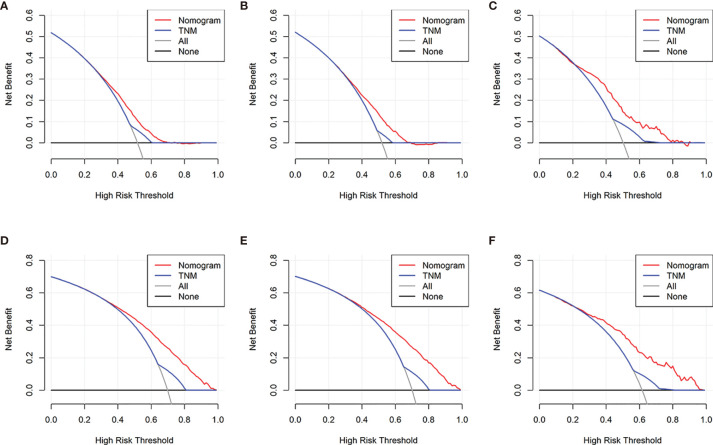
Decision curve analysis of the nomogram and TNM stage for the cancer-specific survival (CSS) and overall survival (OS) prediction of patients with colorectal liver metastasis (CRCLM). **(A)** Training, **(B)** internal validation, and **(C)** external validation cohorts for the CSS; **(D)** training, **(E)** internal validation, and **(F)** external validation cohorts for the OS.

DCA is a novel strategy for evaluating alternative predictive treatment methods and has advantages over AUROC in clinical value evaluation. The DCA curves for the developed nomogram and TNM stage in the training, internal validation, and external validation cohorts are presented in **Figure 8**. Compared with the TNM staging system, the DCA of the nomogram has higher net benefits, indicating that it had better clinical outcome values than TNM stage.

## Discussion

Comprehensive evaluation of the clinicopathological characteristics of patients with CRCLM for developing a corresponding prognosis model is becoming more and more important in terms of prognosis prediction for patients. In this study, a nomogram merging the clinicopathological parameters with the TNM staging system was built to assess the definite 1-, 3-, and 5-year CSS and OS probabilities of CRCLM patients. The characteristics of the nomogram were confirmed by identifying the parameters and it was calibrated, which can contribute to a wide range of applications. From the perspective of ROC curve analysis and detrended correspondence analysis (DCA), the nomogram showed better predictive accuracy and prognostic value compared with the current TNM staging system.

The effect of primary site of CRC on prognosis after CLCLM is still largely unknown. In this study, we found that primary tumor site is closely related to prognosis of patients with CRCLM. In both the training cohort and the internal and external validation cohorts, patients with colorectal primaries had poorer outcomes in OS and CSS than patients with rectal primaries (*P* < 0.0001). The location of primary lesions in the colon was also an independent prognostic factor for patients with CRCLM, similar to previous studies (10). In a retrospective study, it was found that the recurrence-free survival of patients with left-sided primary tumors was shorter than that of patients with right-sided tumors, and the OS of patients with right colorectal cancer after recurrence was shorter (*P* = 0.01) (10). A study determining the influence on CRCLM after portal vein embolization and right hepatic resection and evaluating progression-free survival and prognostic factors for OS found that the location of the primary colorectal cancer was a statistically significant predictor, and right-sided colorectal cancers had a significantly shorter progression-free survival than left-sided colorectal cancers. The presence of extrahepatic disease was associated with poor OS (11). However, a meta-analysis found that the 5-year OS rate of patients with right-sided primary colorectal cancer with liver metastasis after hepatectomy was worse than that of the left-sided primaries (12). Further studies are needed to determine the prognostic impact of the location of the primary tumor in patients with CRCLM.

Lymph node metastasis, total number of lymph nodes obtained, and the number of positive lymph nodes are of prognostic significance in gastrointestinal malignancies. In gastric cancer, the number of acquired lymph nodes less than 16 is an independent adverse prognostic factor (*P* < 0.0001, HR 2.48; 95% CI 1.60–3.70), and the number of acquired lymph nodes may affect the prognosis and staging migration of stage II and stage III gastric cancer patients (13). In a prognostic study of patients with colorectal cancer, the greater the ratio of positive lymph nodes to examined lymph nodes (LNR), the worse the 5-year OS of the patients was (*P* < 0.001), and LNR was shown to be a powerful prognostic factor in colorectal cancer (14). TNM stage, differentiation degree, and lymph node status of the primary tumor are closely related to the prognosis of patients with CRCLM. Studies have shown that patients with resected liver metastasis from colorectal cancer with local lymph node metastasis have a poor survival compared with patients with negative lymph nodes, but a small number of patients with affected lymph nodes can achieve long-term survival (15). The presence of primary lymph node metastasis is associated with prognosis, possibly because the presence of primary lymph node metastasis is a significant risk factor for perihepatic lymph node metastases, while perihepatic lymph node metastases are a negative prognostic factor (16). Zong et al. found that Musashi 2 overexpression, lymph node metastasis, distant metastasis, and TNM stage were associated with liver metastasis of colorectal cancer. Musashi 2 overexpression is associated with poor prognosis and may be a potential biomarker for liver metastasis of colorectal cancer patients (17). In this study, we obtained similar results. Lymph node metastasis and the number of lymph node metastasis have important prognostic significance in patients with CRCLM. In addition, our study also found that several clinicopathological features were closely related to the prognosis of patients with CRCLM, and established the corresponding prognosis model, which could accurately predict their prognosis.

At present, the prognosis prediction models of CRCLM are mainly studied with respect to histopathology and clinicopathology. Liang et al. calculated the immune score based on immunostaining CD3+ and CD8+ cell density and established a predictive model combined with histopathological growth pattern (HGPS) and clinical risk score (CRS), which could be used to stratify the survival of CRCLM patients, but this study lacked internal and external validation (18). Wang et al. established an immune score by detecting the number and density of CD3+ and CD8+ T cells in the tumor center and peritumoral area and predicted the prognosis of recurrence-free survival and OS of CRCLM patients, which was confirmed by internal validation. The results of this study showed that liver metastasis immune score can be used to predict the prognosis of CRCLM patients after hepatectomy (19). Studies have used neutrophil–lymphocyte ratio (NLR), derived NLR, platelet–lymphocyte ratio (PLR), and lymphocyte–monocyte ratio (LMR) inflammation score in resectable CRCLM as prognostic tools. The study found that patients with high preoperative NLR and PLR were independent prognostic factors for CRCLM patients and its prognostic value was superior to other systemic inflammation scores based on cells, but there was no further validation (20, 21). Previous studies used the clinical risk score (CRSS) of Fong and Nordlinger to predict the prognosis of patients with CRCLM (22, 23), but some studies found that CRSS was not a reliable prognostic tool for patients who received neoadjuvant chemotherapy before hepatectomy. Current prognostic models for CRCLM have some deficiencies, mainly due to the limitation of sample size and secondly due to the fact that the indicators are not good enough for accurate prediction. In this study, we analyzed common clinicopathological characteristics, individually and in combination, to establish a convenient, fast, and accurate prediction model. The calculation chart of the calibration curve shows a high degree of credibility.

Our research has some limitations: First, treatment information except for surgery was not available in the SEER database and could not be incorporated into the analysis. Second, in addition to the primary tumor characteristics, tumor location was also related to the location and number of metastatic liver tumors. Because such data were missing in the validation cohort, we could not further analyze the location and number of metastatic tumors in the liver. Third, this was a retrospective study based on limited clinical records, and further prospective multicenter clinical studies are needed to demonstrate the clinical validity of the model. However, this study also has significant advantages. We used routinely available clinicopathological data to establish a convenient, rapid, and accurate prediction model; secondly, this study is a retrospective analysis of a large sample, and internal and external validation queues were used to verify the prediction model to ensure reliability. A relatively large retrospective cohort analysis (large sample size) and a broad meta-analysis of various factors that may be associated with colorectal cancer OS and CSS should provide an important reference point for clinicians.

In conclusion, we established and validated a nomogram based on significant clinicopathological characteristics for predicting CSS and OS in CRCLM patients. The novel nomogram has sufficient discriminatory and calibration capability in addition to exceptional clinical effectiveness and could be an easy-to-use tool for clinicians to promote a personalized postoperative prognostic assessment and further identify treatment strategies for CRCLM patients.

## Data Availability Statement

The original contributions presented in the study are included in the article/**Supplementary Material**. Further inquiries can be directed to the corresponding authors.

## Ethics Statement

The studies involving human participants were reviewed and approved by the Ethical Committee and Institutional Review Board of Wuhan Union Medical College. The patients/participants provided their written informed consent to participate in this study. Written informed consent was obtained from the individual(s) for the publication of any potentially identifiable images or data included in this article.

## Author Contributions

KW, NZ, and KC conceived and designed the study. YC, SK, SD, LY, JG, FM, YX, CZ, WC, HLL, HL, FS, and ZS collected and analyzed the data. YC, SK, and SD wrote the paper. KW, NZ, and KC reviewed and edited the manuscript. All authors contributed to the article and approved the submitted version.

## Funding

This study was supported by grants from the Wuhan High Magnetic Field Interdisciplinary Fund (WHMFC202113) and Free Innovation Pre-Research Fund and Platform Scientific Research Fund in 2019 (02.03.2019-111) and 2020 (F016.02004.20001.023).

## Conflict of Interest

The authors declare that the research was conducted in the absence of any commercial or financial relationships that could be construed as a potential conflict of interest.

## Publisher’s Note

All claims expressed in this article are solely those of the authors and do not necessarily represent those of their affiliated organizations, or those of the publisher, the editors and the reviewers. Any product that may be evaluated in this article, or claim that may be made by its manufacturer, is not guaranteed or endorsed by the publisher.

## Supplementary Material

Supplementary Figure 1 The metastatic surgery indicators were statistically associated with OS and CSS in CRCLM. **(A, B)** The CSS in SEER data and in external validation cohorts; **(C, D)** The OS in SEER data and in external validation cohorts.
