# Supplementary figures and images for: Development and Validation of a Predictive Scoring System for Colorectal Cancer Patients With Liver Metastasis: A Population-Based Study
Source: Front Oncol. 2021 Dec 1;11:719638. doi: 10.3389/fonc.2021.719638 (PMC8671306; doi:10.3389/fonc.2021.719638)

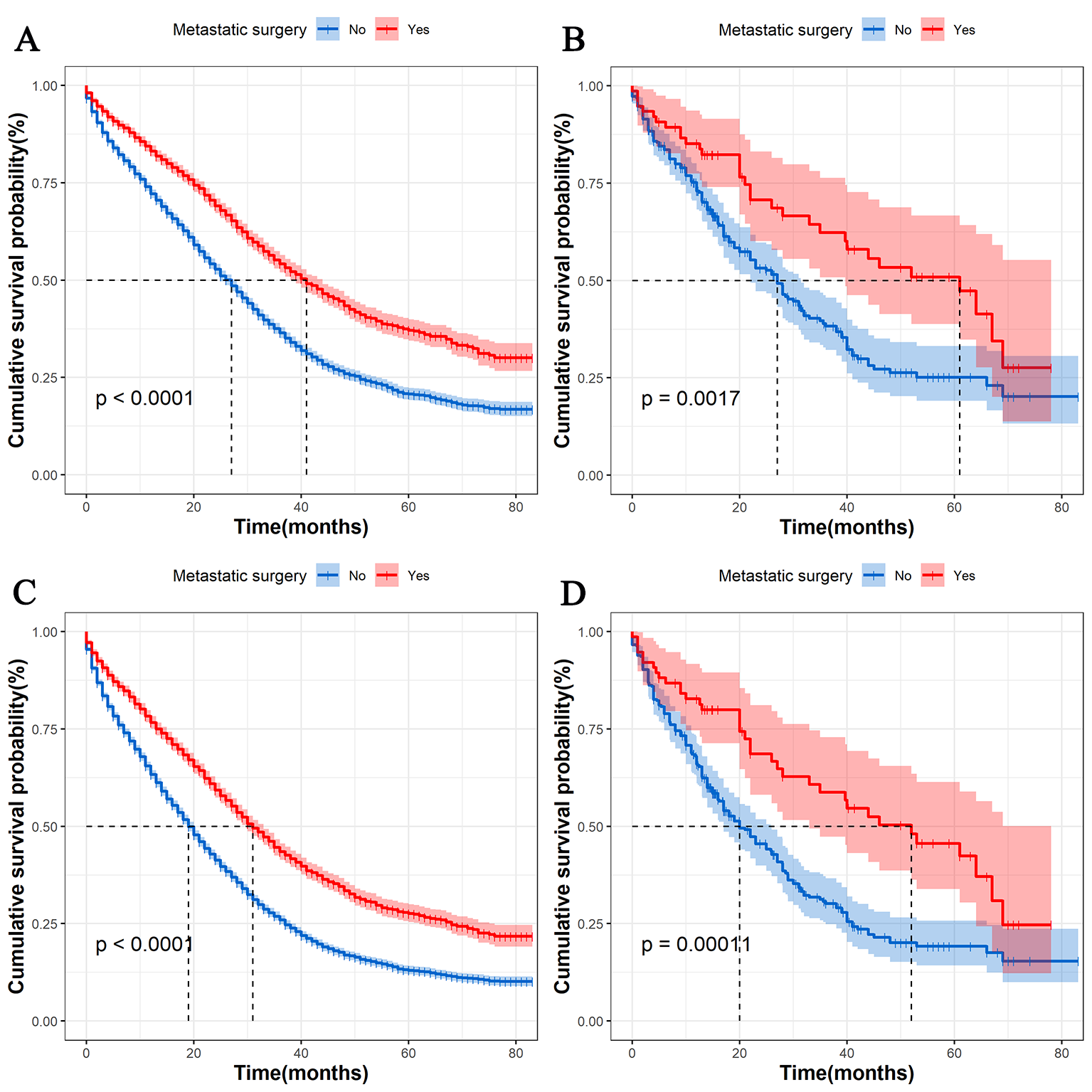

Supplement: Supplementary Figure 1 — The metastatic surgery indicators were statistically associated with OS and CSS in CRCLM. (A, B) The CSS in SEER data and in external validation cohorts; (C, D) The OS in SEER data and in external validation cohorts. [file Image_1.tif]
