# Supplementary material for: Development and Validation of a Predictive Scoring System for Colorectal Cancer Patients With Liver Metastasis: A Population-Based Study
Source: Front Oncol. 2021 Dec 1;11:719638. doi: 10.3389/fonc.2021.719638 (PMC8671306; doi:10.3389/fonc.2021.719638)
Supplement: Supplementary file 2 [file Table_1.doc]

**Supplementary table**

| **Author** | **Country** | **Year** | **Total patients** | **Training cohort** | **Validation cohort** | **Research type** |
| --- | --- | --- | --- | --- | --- | --- |
| Meng et al ^1^ | China | 2021 | 234 | 174 | 60 | Single-center study |
| Tang et al ^2^ | China | 2021 | 203,998 | 203,998 | No | SEER database |
| Li et al ^3^ | China | 2020 | 100 | 100 | No | Single-center study |
| Li et al ^4^ | China | 2020 | 9,958 | 9,958 | No | SEER database |
| Wang et al ^5^ | China | 2018 | 96 | 71 | 25 | Single-center study |
| Liu etal ^6^ | China | 2019 | 564 | 447 | 117 | Single-center study |
| Nathan et al ^7^ | USA | 2010 | 949 | 949 | No | International Multi-Institutional |
| Mo et al ^8^ | China | 2020 | 142343 | 142343 | No | SEER database |
| Yao et al ^9^ | China | 2021 | 241 | 241 | No | Single-center study |
| Beppu ^10^ | Japan | 2012 | 727 | 727 | No | Multicenter study |

1.Meng Q, Zheng N, Wen R, Sui J, Zhang W. Preoperative nomogram to predict survival following colorectal cancer liver metastasis simultaneous resection. J Gastrointest Oncol. 2021 Apr;12(2):556-567. doi: 10.21037/jgo-20-329.

2.Tang M, Wang H, Cao Y, Zeng Z, Shan X, Wang L. Nomogram for predicting occurrence and prognosis of liver metastasis in colorectal cancer: a population-based study. Int J Colorectal Dis. 2021 Feb;36(2):271-282. doi: 10.1007/s00384-020-03722-8.

3.Li M, Li X, Guo Y, Miao Z, Liu X, Guo S, Zhang H. Development and assessment of an individualized nomogram to predict colorectal cancer liver metastases. Quant Imaging Med Surg. 2020 Feb;10(2):397-414. doi: 10.21037/qims.2019.12.16.

4.Li Y, Liu W, Zhao L, Güngör C, Xu Y, Song X, Wang D, Zhou Z, Zhou Y, Li C, Pei Q, Tan F, Pei H. Nomograms predicting Overall Survival and Cancer-specific Survival for Synchronous Colorectal Liver-limited Metastasis. J Cancer. 2020 Aug 27;11(21):6213-6225. doi: 10.7150/jca.46155.

5.Wang Y, Zheng J, Chen H, Hu C, Sun B, Wang H, Shi Q, Long J, Zhang H, Li W. A prognostic nomogram for colorectal cancer liver metastases after percutaneous thermal ablation. Int J Hyperthermia. 2018 Sep;34(6):853-862. doi: 10.1080/02656736.2017.1368095.

6.Liu W, Wang K, Han Y, Liang JY, Li YH, Xing BC. Nomogram predicted disease free survival for colorectal liver metastasis patients with preoperative chemotherapy followed by hepatic resection. Eur J Surg Oncol. 2019 Nov;45(11):2070-2077. doi: 10.1016/j.ejso.2019.06.033.

7.Mo S, Cai X, Zhou Z, Li Y, Hu X, Ma X, Zhang L, Cai S, Peng J. Nomograms for predicting specific distant metastatic sites and overall survival of colorectal cancer patients: A large population-based real-world study. Clin Transl Med. 2020 Jan;10(1):169-181. doi: 10.1002/ctm2.20.

8.Nathan H, de Jong MC, Pulitano C, Ribero D, Strub J, Mentha G, Gigot JF, Schulick RD, Choti MA, Aldrighetti L, Capussotti L, Pawlik TM. Conditional survival after surgical resection of colorectal liver metastasis: an international multi-institutional analysis of 949 patients. J Am Coll Surg. 2010 May;210(5):755-64, 764-6. doi: 10.1016/j.jamcollsurg.2009.12.041.

9.Yao J, Chen Q, Deng Y, Zhao J, Bi X, Li Z, Huang Z, Zhang Y, Zhou J, Zhao H, Cai J. Nomograms predicting primary lymph node metastases and prognosis for synchronous colorectal liver metastasis with simultaneous resection of colorectal cancer and liver metastases. Ann Palliat Med. 2021 Apr;10(4):4220-4231.

10.Beppu T, Sakamoto Y, Hasegawa K, Honda G, Tanaka K, Kotera Y, Nitta H, Yoshidome H, Hatano E, Ueno M, Takamura H, Baba H, Kosuge T, Kokudo N, Takahashi K, Endo I, Wakabayashi G, Miyazaki M, Uemoto S, Ohta T, Kikuchi K, Yamaue H, Yamamoto M, Takada T. A nomogram predicting disease-free survival in patients with colorectal liver metastases treated with hepatic resection: multicenter data collection as a Project Study for Hepatic Surgery of the Japanese Society of Hepato-Biliary-Pancreatic Surgery. J Hepatobiliary Pancreat Sci. 2012 Jan;19(1):72-84. doi: 10.1007/s00534-011-0460-z.
